# Searching for signatures of sexually antagonistic selection on stickleback sex chromosomes

**DOI:** 10.1098/rstb.2021.0205

**Published:** 2022-08-01

**Authors:** Andrius J. Dagilis, Jason M. Sardell, Matthew P. Josephson, Yiheng Su, Mark Kirkpatrick, Catherine L. Peichel

**Affiliations:** ^1^ Department of Integrative Biology, University of Texas, Austin TX 78712, USA; ^2^ Department of Computer Science, University of Texas, Austin TX 78712, USA; ^3^ Department of Biology, University of North Carolina, Chapel Hill, NC 27599, USA; ^4^ Division of Evolutionary Ecology, Institute of Ecology and Evolution, University of Bern, 3012 Bern, Switzerland

**Keywords:** sex chromosome evolution, sexually antagonistic selection, Japan Sea stickleback, supergenes

## Abstract

Intralocus sexually antagonistic selection occurs when an allele is beneficial to one sex but detrimental to the other. This form of selection is thought to be key to the evolution of sex chromosomes but is hard to detect. Here we perform an analysis of phased young sex chromosomes to look for signals of sexually antagonistic selection in the Japan Sea stickleback (*Gasterosteus nipponicus*). Phasing allows us to date the suppression of recombination on the sex chromosome and provides unprecedented resolution to identify sexually antagonistic selection in the recombining region of the chromosome. We identify four windows with elevated divergence between the X and Y in the recombining region, all in or very near genes associated with phenotypes potentially under sexually antagonistic selection in humans. We are unable, however, to rule out the alternative hypothesis that the peaks of divergence result from demographic effects. Thus, although sexually antagonistic selection is a key hypothesis for the formation of supergenes on sex chromosomes, it remains challenging to detect.

This article is part of the theme issue ‘Genomic architecture of supergenes: causes and evolutionary consequences’.

## 1. Introduction

In many taxa, non-recombining regions of sex chromosomes, termed the sex-determining region (SDR), act as supergenes [1]. Supergenes are linked loci that are inherited as a single locus and generate alternative phenotypes [2], properties common to the SDR. The mammalian Y, for example, has a large non-recombining SDR and carries multiple genes necessary for male development and fertility [3]. The boundaries of the SDR are not static, and distinct evolutionary ‘strata’ can form as recombination is sequentially suppressed across the chromosome [1,4,5]. It is unclear, however, why the SDR expands in some taxa but not others.

The leading hypothesis for the origin and later expansion of the SDR is sexually antagonistic selection (SAS) [6]. SAS occurs when an allele is beneficial in one sex but deleterious in the other [7]. Males and females frequently have different fitness optima, which results in SAS that drives the evolution of sexual dimorphism [8] and sex-biased gene expression [9]. While the SDR carries alleles that are restricted to the X or the Y (or Z and W in ZW systems), polymorphisms under SAS can accumulate in the recombining pseudoautosomal region (PAR) of sex chromosomes [10–12]. These can then drive the suppression of recombination between X and Y chromosomes by linking male beneficial alleles to the sex-determining locus on the Y chromosome, which prevents the deleterious fitness effects that occur when those alleles are carried by females [1,13].

The observation of genes with sex-specific fitness effects in regions of reduced recombination in or near the SDR is consistent with that idea, but there is a strong alternative hypothesis: polymorphisms subject to SAS can accumulate after recombination is suppressed [14,15]. This possibility is supported by examples of male-beneficial genes that have been transposed from autosomes to the SDR of the sex chromosomes of *Drosophila* [16–21], mammals [22,23], and the threespine stickleback [24]. The second alternative hypothesis is even simpler—some sex chromosomes may simply never accrue loci under SAS in a fashion disproportionate to the rest of the genome [25]. Indeed, recent work has demonstrated that population genetic approaches may be biased to identifying SAS on the X [26], and there have been several calls to search for alternative explanations for the evolution of sex chromosomes in general [27,28]. Finding evidence of SAS in PARs is therefore a key goal of sex chromosome research.

Models show that polymorphisms under SAS in the PAR will generate characteristic patterns of molecular variation that can be more conspicuous than those on autosomes or in the SDR [29]. Because sites under SAS on autosomes are unlinked to the SDR, any build up in linkage disequilibrium (LD) between loci under SAS and the sex chromosomes is erased in each generation. SAS can still be detected as minor allele frequency differences between males and females (e.g. [30,31]), but these signals are quite weak. On the other hand, the PAR is linked to the SDR and LD can build up over multiple generations, leading to a stronger signal of SAS. In particular, the X and Y are expected to show peaks of differentiation around sites under SAS, allowing them to be mapped with higher resolution than is possible elsewhere in the genome. Allele frequency differences between the sexes in the PARs of the white campion [32,33] and hops [34] are consistent with SAS, but it has not been possible to rule out alternative hypotheses. Guppies show broad peaks of divergence between males and females in regions of the PAR with very low recombination [35], but these span large numbers of genes.

The sex chromosomes of the Japan Sea stickleback (*Gasterosteus nipponicus*) are highly suited to studies of sexually antagonistic selection. The Japan Sea stickleback diverged from its sister species, the threespine stickleback (*Gasterosteus aculeatus*), between 0.6 and 1.2 Ma [36]. Sex is determined by chromosome 19 (Chr 19) in both species [37–39]. The threespine stickleback has a PAR of 2.5 Mb and a non-recombining SDR of 17.5 Mb that contains three evolutionary strata that correspond to three inversions [24,38]. In the Japan Sea stickleback, the ancestral Y on Chr 19 fused with an autosome, chromosome 9 (Chr 9), to produce a ‘neo-Y’ chromosome [39,40] (electronic supplementary material, figure S1a). The unfused homologue of the neo-Y co-segregates with the ancestral X, and so is referred to as a ‘neo-X’. A large (7 Mb) region of the neo-Y adjacent to the fusion has ceased to recombine, resulting in an expanded SDR [39–41]. The remaining 13.7 Mb of Chr 9 continues to recombine as a second PAR. A quantitative trait locus (QTL) study of Japan Sea stickleback mapped two chromosome regions affecting phenotypes involved with courtship (and so are potential targets of SAS) to the PAR on Chr 9 [39].

In prior work, we phased the entire genome of the Japan Sea stickleback using a small pedigree design (electronic supplementary material, figure S1b) [42], giving us phased X and Y sequences. This significantly increases our power to detect differences between the X and Y relative to the more common strategy of indirectly comparing the sex chromosomes by comparing XY males against XX females (e.g. [32,34,35]). Here, we first use these phased data to describe patterns of divergence and diversity on the sex chromosomes of the Japan Sea stickleback. We then look for windows with elevated X-Y divergence, which would be consistent with evidence of SAS in the PAR of its neo-sex chromosome pair.

## 2. Methods

### (a) Sampling and crosses

We sampled 15 Japan Sea males and 13 threespine females from Akkeshi Bay and Biwase on Hokkaido, Japan. To obtain phased X and Y sequences, 15 hybrid crosses were made from these individuals, using one threespine female three times. DNA was extracted from fin clips taken from each father and mother, plus one son and one daughter per cross (electronic supplementary material, figure S1b). Paired end whole-genome sequencing was conducted on all 58 individuals. Eight crosses were sequenced at the Genomics Shared Resources of the Fred Hutchinson Cancer Research Center on an Illumina HiSeq 2500, and the remainder were sequenced at the Next Generation Sequencing Platform of the University of Bern on an Illumina HiSeq 3000. The average coverage was 28X. Full details of the crosses and sequencing are given in Sardell *et al.* [42].

### (b) Genotyping and phasing

We used *FastQC* v. 11.5 [43] to remove poor quality reads. Reads were mapped to the Glazer *et al.* [44] threespine stickleback reference genome using *bwa mem* v. 7.12 [45]. SNPs were sorted using *SAMtools*, per sample depth was calculated using *SAMtools depth*, and genotypes were called using *mpileup* v. 1.3 [46]. We filtered variants for minimum quality scores of 999, a minimum genotype quality of 20, and a minimum mean depth of 10X using *VCFTools* v. 1.15 [47]. To reduce genotyping errors resulting from mapping of paralogues, we also removed variants with mean depth greater than 1.5 times the median coverage on Chr 9 and 19. Lastly, variants were filtered to biallelic single nucleotide polymorphisms (SNPs) using *VCFTools* v. 1.15.

We used a custom *R* script (previously made available in [48]) to phase the data. Briefly, at all heterozygous sites in an offspring, we identified the paternal allele as that which was present in the father and absent in the mother. Crossing different species gave us increased power to phase, as the parents were fixed for alternate variants for many of the heterozygous sites in the offspring. We removed any sites in which the offspring and both parents were all heterozygous because they cannot be phased. Sites at which an offspring is homozygous for an allele that is not carried by one of its parents were discarded as they result from sequencing or other type of error. We filtered out any sites where more than five offspring across all families were missing phased genotypes. This pipeline resulted in phased sequences of the sex chromosomes, with 112 463 SNPs on Chr 9 and 81 061 SNPs on Chr 19 across the paternally inherited sex chromosomes from Japan Sea stickleback and the maternally inherited sex chromosomes from threespine stickleback. Of these, 35 409 sites on Chr 9 and 58 284 sites on Chr 19 were polymorphic among the 15 X chromosomes and 15 Y chromosomes sampled from the Japan Sea stickleback.

Population genetic analyses used to describe the evolution of the Japan Sea sex chromosome are detailed in the electronic supplementary material. Briefly, we calculated divergence (*K*) between the threespine and Japan Sea stickleback on Chr 9 and Chr 19. We also calculated divergence (*K*), nucleotide diversity (*π*) and Tajima's *D* between the Japan Sea X and Y in 10 kb windows. On average, we observed 10 SNPs per 10 kb window across the PAR on Chr 9. We therefore built gene trees in 100 kb non-overlapping windows to have a larger number of phylogenetically informative sites. In the recombining PAR, the X and Y are intermingled on the gene trees, while in the non-recombining SDR, all Y chromosomes form a monophyletic clade to the exclusion of the X chromosomes [48,49]. Gene trees with this property are said to be ‘XY gene tree consistent’. Finally, we calculated *d*_n_, *d*­_s_ and *d*_n_/*d*_s_ on the Japan Sea X and the Y relative to the threespine stickleback using gene annotations from Glazer *et al.* [44].

### (c) Detecting sexually antagonistic selection

The region on the PAR close to the SDR is an attractive region to search for signals of SAS using the phased sequencing data from the Japan Sea X and Y chromosomes. This is because peaks of *F*_ST_ resulting from SAS are more visible on the PAR [29]. However, in the region of the PAR that is extremely close to the SDR (within several *ρ*, where *ρ* = 4*N*_e_ *r*, *N*_e_ is the effective population size, and *r* is the rate of recombination in males between the focal site and the SDR), high *F*_ST_ between the X and the Y is expected even in the absence of SAS, owing to LD between the site and the SDR. To determine the region of the PAR on Chr 9 suitably distant from the SDR, we modified previously published neutral coalescent simulations [29,50] to account for two kinds of demographic effects: the demographic history of the species [36], and the selective sweep that occurred when the neo-Y was established. For the latter, we assumed the neo-Y originated just after the Japan Sea and threespine sticklebacks diverged 306 000 generations ago [36]. (Further details are given in the electronic supplementary material, and the code is available at https://github.com/adagilis/SexCoal). We simulated 10^4^ replicates, each producing samples of sequences from 15 X and 15 Y chromosomes. Low density linkage maps [39,41,42] and coalescent simulations (electronic supplementary material, figure S2) suggest that the region in the PAR of Chr 9 starting at 7.5 Mb, which lies at 0.6 Mb (= 0.6 cM = 60 *ρ*) from the SDR, has sufficiently high recombination with the SDR to avoid high *F*_ST_ in the absence of SAS. While windows as close as 40 *ρ* show reduced *F*_ST_, we conservatively chose a 50% greater distance given the uncertainty in recombination rates within the region. There are also reasons to limit how far from the SDR we should seek signals of SAS. SAS is less likely to maintain polymorphisms when recombination with the SDR is high and correcting for more multiple comparisons takes an increasing toll on statistical power. We therefore defined a focal region for the search for SAS starting at 7.5 Mb and ending at 11.5 Mb (roughly 460 *ρ* from the SDR).

Theory shows that a peak in *F*_ST_ caused by SAS is expected to be several *ρ* wide [29]. We estimate that *ρ* corresponds to about 10 kb in the Japan Sea stickleback, given estimates for *N_e_* of 10^5^ [36] and a local recombination rate of 1 cM/Mb on Chr 9 in males near the SDR [41]. We therefore estimated *F*_ST_ between the X and Y chromosomes in non-overlapping windows of 10 kb. A total of 178 10 kb windows meeting our filtering criteria (greater than 10 sites polymorphic among Japan Sea haplotypes) within the focal region of Chr 9 were used for further analysis.

We first used a model-free approach to detect SAS in the focal region. We identify potential SAS targets as windows with higher *F*_ST_ than expected by chance. To generate a null distribution for *F*_ST_, in each 10 kb window we randomized the labels of X and Y haplotypes, keeping equal numbers of Xs and Ys, 10^6^ times. The *p*-value for each window was calculated as the proportion of randomizations for that window with an *F*_ST_ value equal to or greater than the observed value. To correct for multiple comparisons, we used the *qvalue* package in *R*, which returns adjusted *p*-values, following Storey & Tibshirani [51]. Since we are only testing 178 windows and only expected a few outliers at most, we chose a less conservative false discovery rate (FDR) of 0.2 [52].

We used a second approach to look for SAS that incorporates the effects of demographic history and the selective sweep of the Y in generating high *F*_ST_. We first performed 1000 coalescent neutral simulations, each yielding 15 X and 15 Y sequences, under the demographic assumptions described above. To obtain an FDR of 0.2, we used a more stringent multiple comparison correction and adjusted the critical *p*-value so that at least one significant peak of *F*_ST_ was obtained in 20% of the simulations. Further details are given in the electronic supplementary material.

Lastly, we implemented two further approaches to detect SAS: approximate Bayesian computation, and a random forest algorithm. Further details and justifications are provided in the electronic supplementary material. Neither approach could reliably distinguish between PARs containing loci with SAS and those without.

## 3. Results and discussion

### (a) Evolution of the Japan Sea sex-determining region

**Figure 1.**
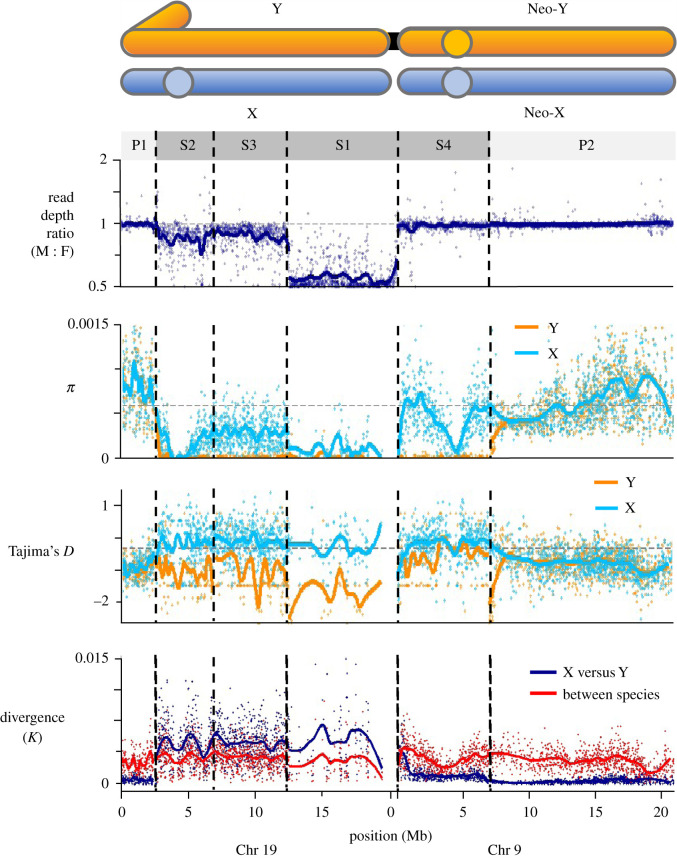
Population genomics of the X and Y chromosomes in Japan Sea stickleback*.* Four statistics are shown for the Japan Sea stickleback sex chromosomes. Dashed vertical lines give the boundaries of the evolutionary strata. Circles in the chromosome schematics show the locations of centromeres. The two PARs are labelled as P1 and P2, and the four strata in the SDR of the Y chromosome are S1, S2, S3 and S4 (from oldest to youngest). Dashed horizontal lines indicate autosomal averages. Loess curves were fitted separately for each PAR and stratum. Read depth ratio was calculated between sons and daughters, while π and Tajima's *D* were calculated for the Japan Sea X and Y. Divergence (*K*) was calculated between the Japan Sea X and Y and between the Japan Sea and threespine haplotypes.

On the ancestral sex chromosome (Chr 19), we find that divergence (*K*) between the X and Y is higher than divergence between the Japan Sea and threespine sticklebacks along the entirety of the SDR shared with threespine stickleback (figure 1). These data are consistent with previous work showing that the SDR comprises three strata that are older than the divergence between Japan Sea and threespine stickleback [36], suggesting the ancestral sex chromosome is shared between species. We therefore define three strata on the ancestral sex chromosome, S1, S2 and S3, at boundaries identified by Peichel *et al.* [24]. We find significant departures from equal read depths in males and females. The read depth ratio is about one half in stratum 1, which is the oldest, and jumps to much higher values in strata 2 and 3 (figure 1). A ratio of one half is expected when much of the Y has been deleted or has diverged to the point that reads from it no longer map to the reference sequence [53]. In the threespine stickleback, 82% of genes have been deleted or have highly degenerated in this stratum of the Y [24,38,54–56], and because of its shared ancestry with the Japan Sea Y it is likely that a similar situation also occurs in that species.

Because stratum 1 is so degenerate, some aspects of its molecular variation are difficult to interpret. We find elevated *F*_ST_ between the X and Y, despite the lack of significant difference in d_N_/d_S_, with substitutions measured relative to the threespine stickleback (electronic supplementary material, figure S3). However, a real difference may be obscured because sequences on the Y that have diverged too much from the X do not map to the reference and so are missing from our data. Both *d*_N_ and *d*_S_ are, however, elevated on the Y compared to the X.

Strata 2 and 3 show less signs of degeneration, but nonetheless show substantial differentiation in allele frequencies (*F*_ST_) between X and Y sequences (electronic supplementary material, figure S4). This may be because they are substantially younger than stratum 1 [24]. Along these strata, the ratio of read depth in males versus females is close to 0.85 (figure 1). The values of *d*_N_, *d*_S_, and *d*_N_/*d*_S_ are all significantly larger on the Y than on the X in this region (electronic supplementary material, figure S3). The lack of degeneration in this region of the ancestral Y contrasts strongly with the closely related blackspotted stickleback (*Gasterosteus wheatlandi*) [48]. In that species, a substantial proportion of the Y on Chr 19 has nearly completely degenerated following a fusion with an autosome, with read depth ratios close to half across all strata of the SDR.

**Figure 2.**
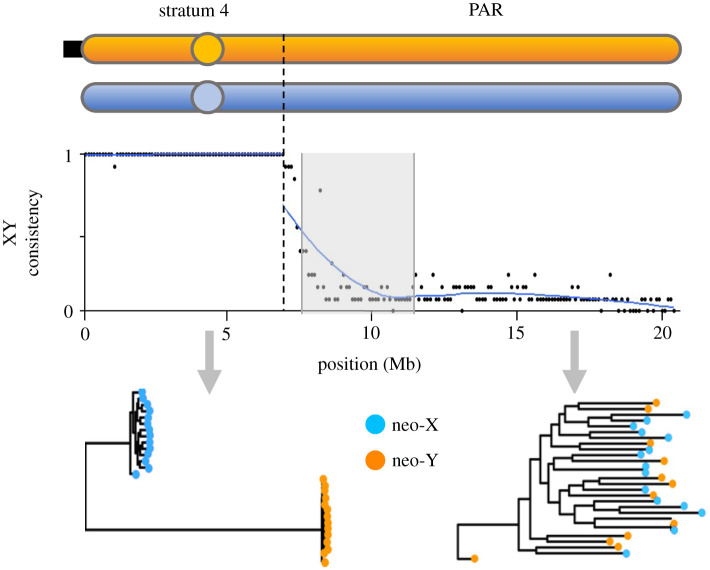
XY gene tree consistency of stratum 4 on Chr 9. XY gene tree consistency maps the boundary between the SDR and PAR to 6.9 Mb (dashed vertical line). The shaded area represents the focal region of the PAR investigated for evidence of SAS (figure 3). At the bottom are examples of a gene tree from the SDR (which shows XY gene tree consistency) and one from the PAR (which does not). Only Japan Sea X and Y data are shown.

Stratum 4, which is on the neo-sex chromosome (Chr 9), is the youngest. To map the boundary between stratum 4 of the SDR and the PAR on the neo-sex chromosome, we used three statistics: divergence (*K*), XY gene tree consistency, and nucleotide diversity on the phased Y chromosome (*π*_Y_). All three statistics place the boundary at the 6.9 Mb position of Chr 9 (figures 1 and 2), which is consistent with earlier findings [40,41].

There is very little evidence of degeneration on the Y along stratum 4: there is no difference in read depth between males and females, and *F*_ST_ is considerably smaller than in strata 1–3 (figure 1, electronic supplementary material, figure S4). *F*_ST_ reaches values close to 1 near the fusion and near the centromere of Chr 9, a pattern driven at least in part by reduced polymorphism on the X (figure 1, electronic supplementary material, figure S4). There is no significant difference in *d*_N_/*d*_S_ between the X and Y in this stratum, although rates of synonymous substitution on the Y are elevated (electronic supplementary material, figure S3).

The divergence (*K*) between the Japan Sea and threespine sticklebacks is much greater than the divergence between the X and Y across much of stratum 4 (figure 1). That suggests that suppression of recombination in this region occurred much more recently than the speciation event (and more recently than suggested by [39]). The speciation event has been dated to between 0.6 and 1.2 million years ago [36], which suggests recombination was lost only within the last few hundred thousand years (or a few hundred thousand generations). It is plausible that recombination ceased in stratum 4 when Chr 9 fused to the S1 end of the ancestral sex chromosome (Chr 19). Consistent with this idea, divergence (*K*) between the X and Y in stratum 4 is highest near to the fusion point, and roughly equal to the divergence between the species (figure 1). This is the pattern expected if recombination was only suppressed in the region immediately adjacent to the fusion when it first appeared. An alternative hypothesis is that the fusion occurred around the time of the speciation event and recombination was suppressed later by some other mechanism. However, we have no evidence of other structural variants on the Japan Sea Y that would have later suppressed recombination [57]. The overall low divergence and degeneration of the Y observed in this region is similar to the neo-sex chromosome of the blackspotted stickleback, in which a fusion occurred between the ancestral Y and Chr 12 [48].

### (b) Signals of sexually antagonistic selection in the pseudoautosomal region

**Figure 3.**
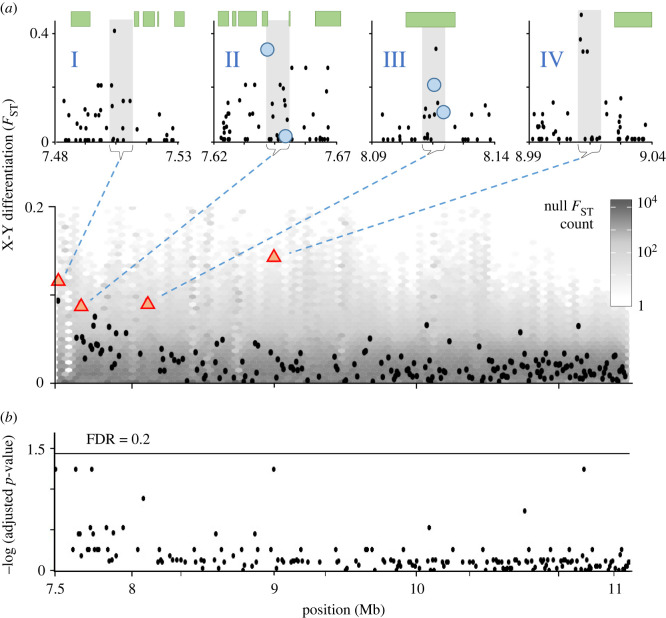
*F*_ST_ peaks between the X and Y in the PAR. (*a*) Bottom: *F*_ST_ between X and Y chromosomes of Japan Sea stickleback in 10 kb windows. The boundary between the SDR and PAR is at 6.9 Mb, to the left of the focal region shown here. The grey heatmap is the null distribution for *F*_ST_ generated by randomizing X and Y labels. Black dots are the average *F*_ST_ for each window. Red triangles are *F*_ST_ values in four windows showing significance with an FDR of 0.2 (for their *F*_ST_ quantiles and *Q*-values see the electronic supplementary material, table S1). Top: the four significant windows for *F*_ST_, showing values of individual SNPs (black dots). The shaded regions show the windows, green rectangles show coding regions for individual genes, and blue circles show nonsynonymous SNPs in coding regions. (*b*) Adjusted *p*-values for each window compared to FDR cut-offs determined by coalescent simulations that incorporate a recent demographic sweep of the Y.

We first used a model-free approach to search for signals of SAS along our focal region of 7.5–11.5 Mb on Chr 9. To evaluate significance, we randomized X and Y labels to generate a null distribution of *F*_ST_ for each window. We find four peaks in *F*_ST_ between the X and Y chromosomes that are significantly greater than expected at an FDR of 0.2 (figure 3; electronic supplementary material, table S1). Under the assumption of independence, the probability of all four peaks are false positives with an FDR of 0.2 is 0.0016. The values of Tajima's *D* among the combined X and Y chromosomes in all four windows are positive (mean = 0.17), while the average for the entire PAR is negative (−0.17). This observation is consistent with SAS acting on sites in those windows. Two of the windows overlap with coding regions, while the other two windows are very close (less than 10 kb) to coding regions. Their proximity to coding regions is not by itself very surprising, however, as more than half of the 178 windows in the PAR fall within that distance of a coding region.

More surprising is that all four of the genes near these *F*_ST_ peaks are associated with mental disorders and neurological development in humans (electronic supplementary material, table S1). Peak I contains an SNP with elevated *F*_ST_ that lies within an exon of a TUBB-like protein, and TUBB is associated with autism and schizophrenia [58]. Peak II is near DNAJB5, a gene implicated in schizophrenia [59]. Peak III overlaps most of the coding region of a gene most similar to human ADD2 and ADD3, associated with bone mineral density [60] and bipolar depression [61], respectively. Two of the SNPs in this peak result in nonsynonymous changes, but neither has a known phenotypic effect. Last, peak IV is upstream of TUSC3, a protein associated with schizophrenia and unipolar depression [62].

Both schizophrenia and depression have been proposed as phenotypes subject to SAS in humans [63]. While we do not know the phenotypic effects of these genes in stickleback, their association with genes for traits potentially under SAS in humans lends further support to the hypothesis that they are under SAS in stickleback. Some 5% of genes in our focal region have orthologues in the human Ensembl GRCh38.p12 database that are included in the gene ontology category ‘nervous system processes’. The probability that four genes chosen at random would be associated with that category is therefore 0.05^4^, or less than 10^−5^. While the proportion of genes that are actually involved in nervous system functions in stickleback is unknown, it does seem plausible that there is a positive association between the windows with high *F*_ST_ between the X and Y in the Japan Sea PAR and nervous system functions subject to SAS.

It is conceivable that one or more of the *F*_ST_ peaks result from errors in which a sequence lying within the SDR is incorrectly mapped to the PAR in the reference genome [64–66]. We evaluated this possibility using windows in the autosomes with *F*_ST_ between paternal haplotypes greater than 0.08, the smallest average value of *F*_ST_ among the four significant windows in the PAR. Based on the frequency of 0.061% of those windows in autosomes, a calculation shows that the probability that mapping errors would cause four or more windows with high *F*_ST_ to occur in the 178 windows of the focal region is less than 10^–5^. Furthermore, no SNPs with high *F*_ST_ in the four candidate windows lie in regions that have high similarity to regions in the SDR, as assessed by blasting 1 kb windows of the peaks against the SDR (although one low *F*_ST_ window in peak I blasts to the SDR). This source of error, therefore, does not seem to account for the patterns seen in the PAR.

It is also possible that the one or more of the *F*_ST_ peaks result from SNPs in LD with the SDR increasing in frequency as the result of demographic effects of the recent population expansion [36] or the selective sweep that occurred when the neo-Y was established. We find that none of our four peaks remain significant when measured against an FDR set by coalescent simulations that include both a sweep of the Y and recent population expansion (figure 3). However, a weakness of this simulation approach is that it relies on several parameters for which we do not have accurate estimates. These include the timing of the sweep on the Y, local recombination rates along the PAR, and recombination rate differences between males and females (see the electronic supplementary material for further details).

Several other caveats apply to our interpretation of the signatures of SAS we find. Despite focusing only on windows with at least 10 high quality SNPs, all four peaks are driven by a small number of SNPs with high *F*_ST_ (figure 3). Peaks I and II could be false positives that show elevated *F*_ST_ simply because of very tight linkage to the SDR, rather than the action of SAS. We estimate that peak I lies about 60 *ρ* from the SDR, which is beyond where the effects of tight linkage to the SDR should affect allele frequencies neutrally evolving chromosomes (electronic supplementary material, figure S2). That estimate depends, however, on estimates of linkage and population size that include uncertainty. Peaks III and IV are much further from the SDR and so are much less likely to result in the absence of SAS. In fact, peak IV is so far away (2.1 cM) that it is somewhat surprising that a signal of SAS is visible there.

## 4. Conclusion

Identifying genes under SAS on sex chromosomes is complex. It is difficult in old sex chromosomes with small PARs, as meiosis requires at least one crossover, and this cross-over occurs in a small region. As a result, the PAR experiences high recombination rates not more conducive to maintaining polymorphisms under SAS than on autosomes [10–12,67–69]. Our study shows that very young PARs also present difficulties. In particular, the effects of demographic history can generate patterns of differentiation between the X and Y that look very much like SAS. Our simulations suggest that vastly increased sample sizes might allow us to distinguish peaks arising from SAS from alternative hypotheses with more certainty. The genes around the peaks in *F*_ST_ we identify make them plausible candidates for SAS, but functional validation is needed to examine whether these polymorphisms underlie fitness differences between males and females to definitively test for SAS. Despite these limitations, the bioinformatics approaches like those in this study are a powerful strategy to identify candidate genes that contribute to the evolution of supergenes.

## Data Availability

All sequence data were previously published in Sardell *et al.* [42] and were previously deposited in the NCBI Short Read Archive, with reference number SRP135745. Coalescent simulation code is available at https://github.com/adagilis/SexCoal and all scripts used to analyse the data are available at https://github.com/adagilis/JapanSeaSAS. Data are provided in the electronic supplementary material [70].
